# Aqueous Extracts of Three Herbs Allelopathically Inhibit Lettuce Germination but Promote Seedling Growth at Low Concentrations

**DOI:** 10.3390/plants11040486

**Published:** 2022-02-11

**Authors:** Kaili Wang, Ting Wang, Cheng Ren, Pengpeng Dou, Zhengzhou Miao, Xiqiang Liu, Ding Huang, Kun Wang

**Affiliations:** 1 College of Grassland Science and Technology, China Agricultural University, Beijing 100193, China; wangkaili@cau.edu.cn (K.W.); wangtingsos@163.com (T.W.); renchengcau@cau.edu.cn (C.R.); pengpengdou@cau.edu.cn (P.D.); 15117111365@163.com (Z.M.); xiqiangliu003@126.com (X.L.); Huangding@263.net (D.H.); 2 Guyuan National Grassland Ecosystem Field Station, Zhangjiakou 076550, China

**Keywords:** allelopathy, *Lactuca sativa*, grassland degradation, leachate, seed germination, seedling growth

## Abstract

Allelopathy is an important process in plant communities. The effects of allelopathy on seed germination and seedling development have been extensively investigated. However, the influences of extract soaking time and concentration on the foregoing parameters are poorly understood. Here, we conducted a seed germination assay to determine the allelopathic effects of the donor herbs *Achnatherum splendens* (Trin.) Nevski, *Artemisia frigida* Willd., and *Stellera chamaejasme* L., from a degraded grassland ecosystem in northern China, on lettuce (*Lactuca sativa* L.) seed germination and early seedling growth. Extract soaking times (12 h or 24 h) did not exhibit significantly different effects on lettuce seed germination or seedling development. However, all aqueous herb extracts inhibited lettuce seed germination and root length (RI < 0) and promoted lettuce shoot length, stem length, leaf length, and leaf width (RI > 0) at both low (0.005 g mL^−1^) and high (0.05 g mL^−1^) concentrations. Moreover, *A. splendens* extracts increased seedling biomass (RI > 0) and synthetical allelopathic effect (SE > 0) at both concentrations. In contrast, both *A. frigida* and *S. chamaejasme* extracts had hormesis effects, which stimulate at low concentrations (RI > 0) but inhibit at high concentrations (RI < 0) on seedling biomass and synthetical allelopathic effect (SE). The results suggest that allelopathic potential may be an important mechanism driving the dominance of *A. frigida* and *S. chamaejasme* in degraded grasslands. Reseeding allelopathy-promoting species such as *A. splendens* may be beneficial to grassland restoration. The present study also demonstrated that seedling biomass, root and shoot length, and seed germination rate are the optimal bioindicators in allelopathy assays and could be more representative when they are combined with the results of multivariate analyses.

## 1. Introduction

Allelopathy is defined as the chemical mediation of plant interactions [1]. The effects of allelopathy on plant invasion, species allocation, community assemblages, forest management, and weed and pest control are being explored in ecological research [2,3,4,5,6,7,8]. Several studies demonstrated that allelopathy affects plant communities and alters interspecific interactions, either by directly affecting plant growth and development or by indirectly affecting decomposition and soil fertility via soil organisms [5,9,10,11,12]. Allelopathy modulates plants by interfering with germination, growth, reproduction, and distribution [13]. The grassland ecosystem is one of the largest in the world; it provides vital services such as livestock forage production and has been the focus of allelopathy studies [14]. Allelopathy and overgrazing have degenerated pastureland [15,16], but recent allelopathy research has focused primarily on invasive plant control, weed management, and herbicide development in cultivated systems [3,6,7]. In contrast, the impact of allelopathy on grassland dynamics has seldom been examined [17]. Inner Mongolia grassland, one of the largest grassland types in China, has been facing the problem of grassland degradation for a long time. Many studies have explored the effects of natural climate change and human disturbance, such as over-grazing [18,19], but have rarely discussed the role of plant interactions in grassland degradation. *Artemisia* *frigida* and *Stellera* *chamaejasme* are grassland degradation bioindicators, as they increase in abundance with grassland degeneration. It has been shown that their allelopathic effects may alter plant community successions [20,21,22,23,24]. Their allelochemicals belong to phenolic compounds [20,24,25]. *Achnatherum splendens* is an herbaceous perennial that is widely distributed in arid and semi-arid areas [26] and predominates in degenerated grasslands. Its congeneric, *A. inebrians,* has allelopathic effects on the growth of other plant species [27,28]. However, little research has been conducted on the allelopathic effects of *A. splendens*.

The digestion, extraction, and concentration of nearly any plant tissue can yield products that are toxic to other plant species [29]. Allelopathy was originally associated with positive, negative, or neutral interactions, but most authors now believe that allelopathic interactions are exclusively negative [30]. Allelopathic effect varies greatly but can reduce the overall plant performance by 25% [31]. However, the mere presence of potentially inhibitory chemicals in plant tissues does not necessarily indicate allelopathy [32]. In fact, the term may be referred to as allelopathic potential based on the description of allelopathic effects observed in laboratory bioassays [17]. Plant secondary metabolites are released into the environment by foliar leaching, volatilization, residue decomposition, and root exudation [1]. Of these, foliar leaching was the most investigated source of allelochemicals [31]. Moreover, leachates from belowground organs and tissues had weaker inhibitory effects than those from aboveground parts [20,31]. Hence, the main method of verifying allelopathic potential is to evaluate the effects of aqueous extracts of donor plant shoots on recipient plants.

Seed germination is vital for the establishment of a stable plant population and is regulated by numerous environmental [33] and biotic factors, such as the allelopathic effects of neighbor plants [20]. Several studies reported that allelopathic compounds extracted from donor plants may significantly reduce seed germination and seedling growth in recipient plants in a concentration-dependent manner [23,34,35,36,37]. Other authors stated that allelochemicals released by donor plants exerted positive effects on recipient plants at low concentrations, but were phytotoxic above certain threshold doses [13,34]. This pattern may be characteristic of the hormesis effect, in which the allelochemical is stimulatory at low concentrations but inhibitory at high concentrations. In practice, however, there is no apparent pattern in terms of the stimulatory, inhibitory, or hormesis effects of different concentrations of plant extracts. In certain studies, the donor plant tissues were soaked at room temperature (25 °C) for 24, 48, or 72 h [38,39,40,41,42]. The authors believed that the quantity of allelochemical extracted would increase with soaking time. However, the risk of mildew formation also increases with soaking time in our former investigation. Hence, the optimal soaking time required for allelochemical extraction remains to be established. In allelopathy bioassay experiments, the test parameters may include seed germination and seedling growth metrics [31]. Various researchers have selected different indices such as germination rate, index, and potential; mean germination time; seedling height (shoot length); root length; leaf length and width; total biomass; root and shoot biomass [34,38,40,42]. However, there is a lack of research on the traits that rapidly indicate recipient plant responses to allelochemicals.

In the present study, we analyzed the influences of leachates secreted by three degraded grassland plant shoots on lettuce (*Lactuca sativa*) seed germination and seedling development. We selected *L. sativa* as the recipient plant, as it is sensitive to allelopathy and has high germination rates and rapid growth [34,43,44]. The aims of this study were to (1) clarify whether aqueous shoot extracts of donor plants have hormesis effects, (2) compare the allelopathic effects among the donor plants, (3) determine whether donor plant soaking time affects allelopathy, and (4) screen suitable germination characteristics of lettuce to evaluate allelopathic potential.

## 2. Results

### 2.1. Effects of Aqueous Herb Extracts on Lettuce Seed Germination

**Figure 1:**
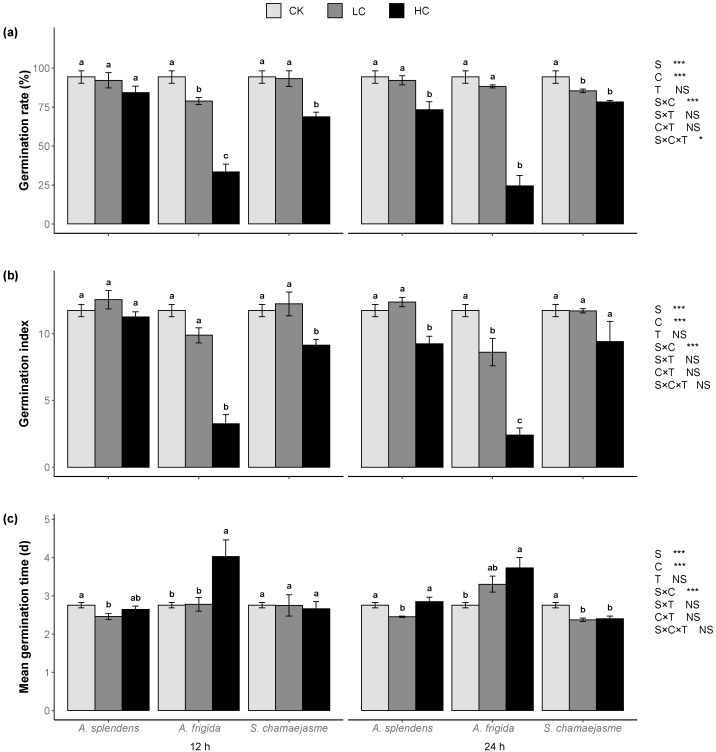
Effects of extract concentration and soaking time of three donor species: *Achnatherum splendens*, *Artemisia frigida*, and *Stellera chamaejasme* on lettuce seed germination rate (**a**), germination index (**b**), and mean germination time (**c**). Data are presented as mean ± SE (*n* = 54). Different letters indicate significant differences among extract concentration by the same donor species and soaking time (*p* = 0.05). CK, control. LC, low concentration (0.005 g mL^−1^). HC, high concentration (0.05 g mL^−1^). S, donor species. C, extract concentration. T, soaking time. * *p* < 0.05, ** *p* < 0.01, *** *p* < 0.001. There were no significant differences between the two soaking times with the Wilcoxon rank sum test (*p* > 0.05).

Plant donor species, extract concentrations, and their interactions significantly affected *L. sativa* seed germination rate, germination index, and mean germination time (Figure 1, *p <* 0.05). However, soaking time had no significant effect on any of the foregoing parameters (Figure 1, *p >* 0.05). S-C-T interactions significantly affected the lettuce seed germination rate (Figure 1, *p <* 0.05). Overall, increasing concentrations of the aqueous extracts of all three donor plants significantly decreased lettuce seed germination rate and germination index (Figure 1a,b, *p <* 0.05). The aqueous *A. frigida* extracts inhibited the foregoing parameters more than those of *A. splendens* or *S. chamaejasme* (Figure 1b). Moreover, the mean lettuce seed germination time increased in response to the aqueous *A. frigida* extracts but decreased in response to those of *A. splendens* and *S. chamaejasme* (Figure 1c).

### 2.2. Effects of Aqueous Herb Extracts on Lettuce Seedling Growth

**Figure 2:**
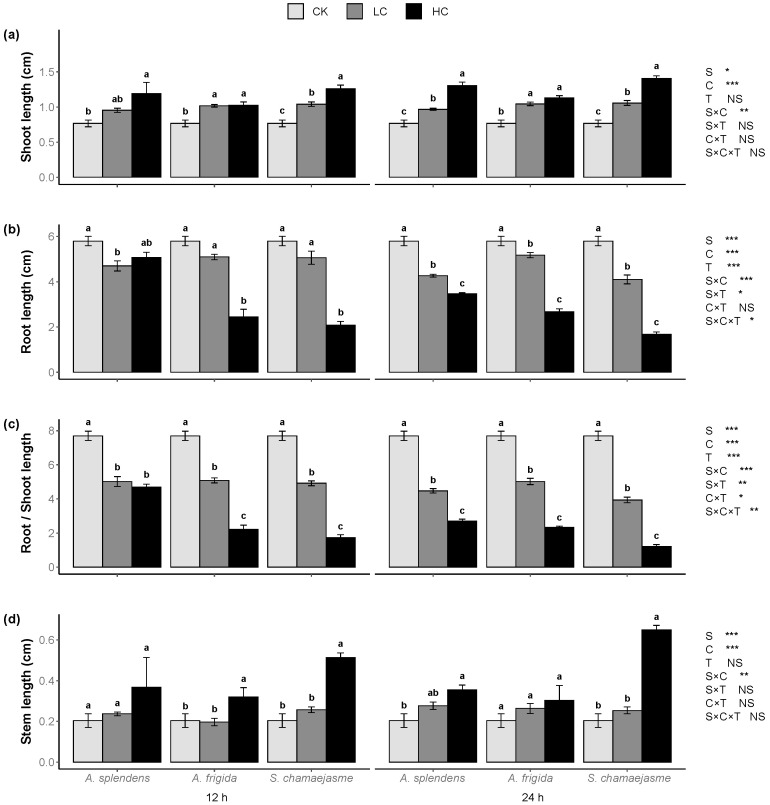
Effects of extract concentration and soaking time of three donor species: *Achnatherum splendens*, *Artemisia frigida*, and *Stellera chamaejasme* on lettuce shoot length (**a**), root length (**b**), root/shoot length (**c**), and stem length (**d**). Data are presented as mean ± SE (*n* = 54). Different letters indicate significant differences among extract concentration by the same donor species and soaking time (*p* = 0.05). CK, control. LC, low concentration about (0.005 g mL^−1^). HC, high concentration (0.05 g mL^−1^). S, donor species. C, extract concentration. T, soaking time. * *p* < 0.05, ** *p* < 0.01, *** *p* < 0.001. There were no significant differences between the two soaking times with the Wilcoxon rank sum test (*p* > 0.05).

**Figure 3:**
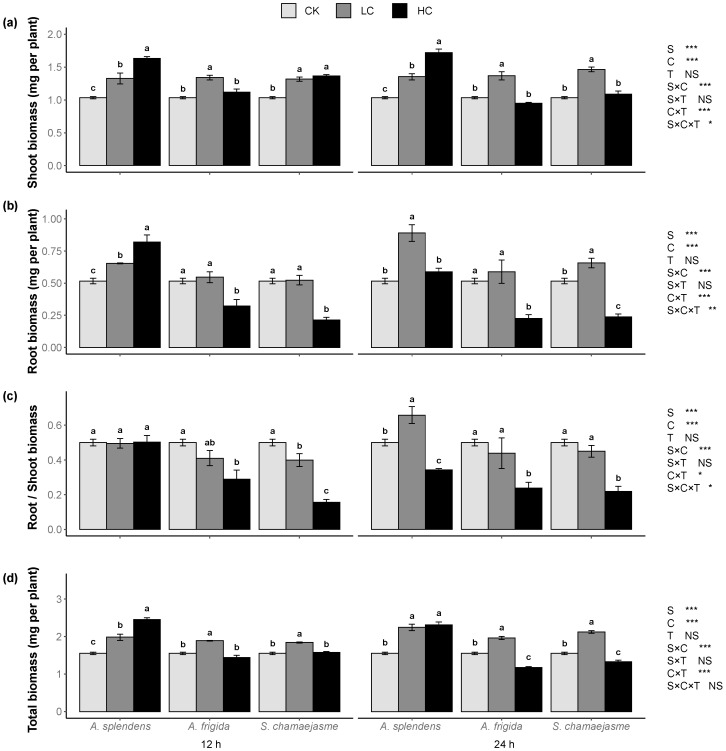
Effects of extract concentration and soaking time of three donor species: *Achnatherum splendens*, *Artemisia frigida*, and *Stellera chamaejasme* on lettuce shoot biomass (**a**), root biomass (**b**), root/shoot biomass (**c**), and total biomass (**d**). Data are presented as mean ± SE (*n* = 54). Different letters indicate significant differences among extract concentration by the same donor species and soaking time (*p* = 0.05). CK, control. LC, low concentration (0.005 g mL^−1^). HC, high concentration (0.05 g mL^−1^). S, donor species. C, extract concentration. T, soaking time. * *p* < 0.05, ** *p* < 0.01, *** *p* < 0.001. There were no significant differences between the two soaking times with the Wilcoxon rank sum test (*p* > 0.05).

**Figure 4:**
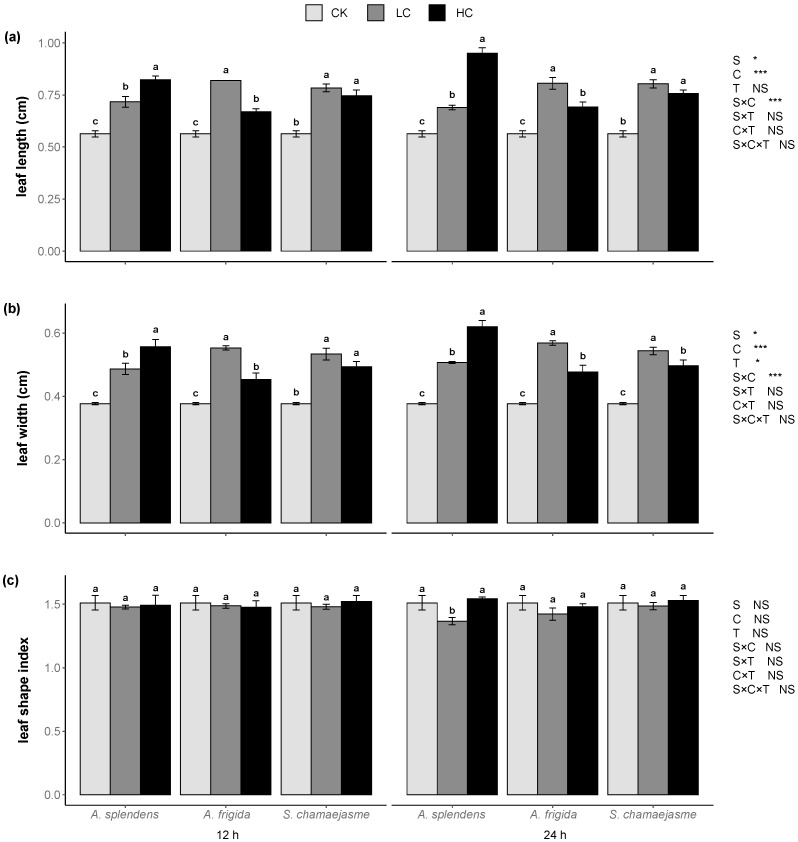
Effects of extract concentration and soaking time of three donor species: *Achnatherum splendens*, *Artemisia frigida*, and *Stellera chamaejasme* on lettuce leaf length (**a**), leaf width (**b**), and leaf shape index (**c**). Data are presented as mean ± SE (*n* = 54). Different letters indicate significant differences among extract concentration by the same donor species and soaking time (*p* = 0.05). CK, control. LC, low concentration (0.005 g mL^−1^). HC, high concentration (0.05 g mL^−1^). * *p* < 0.05, ** *p* < 0.01, *** *p* < 0.001. There were no significant differences between the two soaking times with the Wilcoxon rank sum test (*p* > 0.05).

*L. sativa* seedling development was significantly affected by donor plant species, extract concentrations, and their interactions (Figure 2, Figure 3 and Figure 4, *p <* 0.05). Soaking time had no significant effect on *L. sativa* seedling growth according to Wilcoxon’s rank sum test (Figure 2, Figure 3 and Figure 4, *p >* 0.05). However, the interactions between soaking time and the other factors significantly affected lettuce root length, root/shoot length ratio, shoot biomass, root biomass, root/shoot biomass ratio, and total biomass according to a three-way ANOVA permutation test (Figure 2, Figure 3 and Figure 4, *p <* 0.05). The aqueous extracts of the three herbs significantly enhanced the shoot and stem length in a dose-dependent manner (Figure 2a,d, *p <* 0.05) but significantly inhibited the root length and root/shoot length ratio in a dose-dependent manner (Figure 2b,c, *p <* 0.05). Aqueous extracts of *A. splendens* stimulated the lettuce shoot biomass, root biomass, and total biomass. However, at a high concentration (HC, 0.05 g mL^−1^), it reduced the root/shoot biomass ratio after 24 h of soaking (Figure 3a–d, *p <* 0.05). A low concentration (LC, 0.005 g mL^−1^) of aqueous *A. frigida* and *S. chamaejasme* extracts promoted shoot biomass and had hormesis effects on root and total biomass, while a high concentration (HC, 0.05 g mL^−1^) inhibited the root/shoot biomass ratio (Figure 3a–d, *p <* 0.05). Aqueous extracts of all three herbs significantly stimulated lettuce leaf length and width (Figure 4a,b, *p <* 0.05), to a greater extent at low than high concentration. The aqueous herb extracts did not affect the lettuce leaf shape index. However, the *A. splendens* extract reduced this parameter after 24 h soaking (Figure 4c).

### 2.3. Allelopathic Potential of Aqueous Herb Extracts on Lettuce Seed Germination

**Figure 5:**
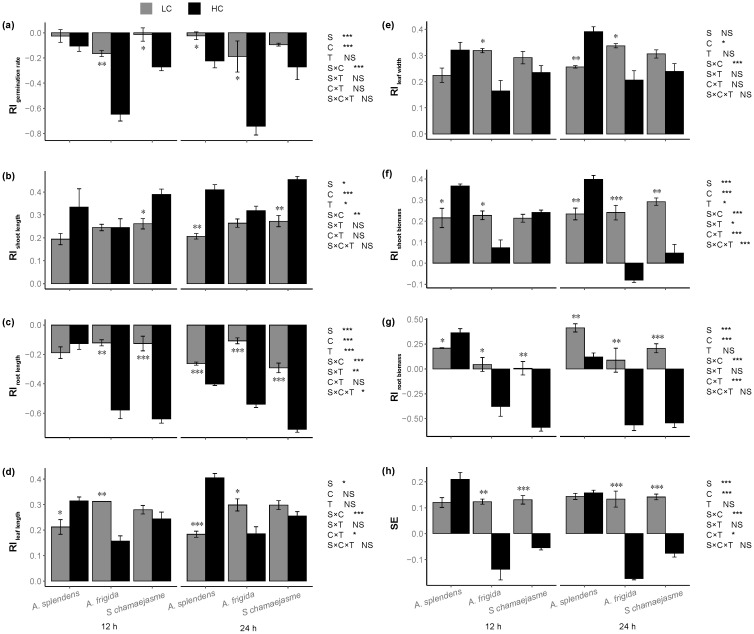
Effects of extract concentration and soaking time of three donor species: *Achnatherum splendens*, *Artemisia frigida*, and *Stellera chamaejasme* on lettuce RI_germination rate_ (**a**), RI_shoot length_ (**b**), RI_root length_ (**c**), RI_leaf length_ (**d**), RI_leaf width_ (**e**), RI_shoot biomass_ (**f**), RI_root biomass_ (**g**), and SE (**h**). Data are presented as mean ± SE (*n* = 36). LC, low concentration (0.005 g mL^−1^). HC, high concentration (0.05 g mL^−1^). S, donor species. C, extract concentration. T, soaking time. * *p* < 0.05, ** *p* < 0.01, *** *p* < 0.001. There were no significant differences between the two soaking times with the Wilcoxon rank sum test (*p* > 0.05).

Donor plant species, extract concentrations, and their interaction significantly affected *L. sativa* allelopathy response index (RI) (Figure 5, *p <* 0.05). Soaking time had no significant effect on *L. sativa* seedling growth according to Wilcoxon’s rank sum test (Figure 5, *p >* 0.05). In contrast, the interactions between soaking time and the other treatments significantly affected lettuce RI_oot length_, RI_shoot biomass_, RI_root biomass_, RI_leaf length_, and SE according to a three-way ANOVA permutation test (Figure 5, *p <* 0.05). All three aqueous herb extracts inhibited lettuce germination rate and root length (RI < 0); the inhibition was relatively stronger at high concentration (HC, 0.05 g mL^−1^) than that at low concentration (LC, 0.005 g mL^−1^) (Figure 5a,c, *p <* 0.05). However, the inhibitory effects of the *A. splendens* extract did not significantly differ between the two concentrations after 12 h soaking (Figure 5a,c). In contrast, all three herb extracts promoted lettuce shoot length and biomass and leaf length and width (RI > 0). However, the HC aqueous *A. frigida* extract inhibited these parameters after 24 h soaking (Figure 5b,d–f). The *A.* *frigida* and *S. chamaejasme* aqueous extracts promoted lettuce shoot length in a concentration-dependent manner (Figure 5b). By contrast, their stimulatory effects on lettuce leaf length and width and shoot biomass decreased with increasing concentration (Figure 5d–f). Moreover, the *A. splendens* aqueous extract promoted lettuce shoot and leaf length and leaf width in a concentration-dependent manner (Figure 5b–e). Aqueous *A.* *splendens* extract promoted lettuce root biomass, whereas those of *A. frigida* and *S. chamaejasme* showed hormesis effects (Figure 5g, *p <* 0.05). Overall, the SE indicated that *A. splendens* stimulated lettuce seed germination and seedling growth while *A. frigida* and *S. chamaejasme* had hormesis effects on these parameters (Figure 5h, *p <* 0.05). Furthermore, *A. frigida* was more inhibitory than *S. chamaejasme* (Figure 5h).

### 2.4. Vital Germination Characteristics

**Figure 6:**
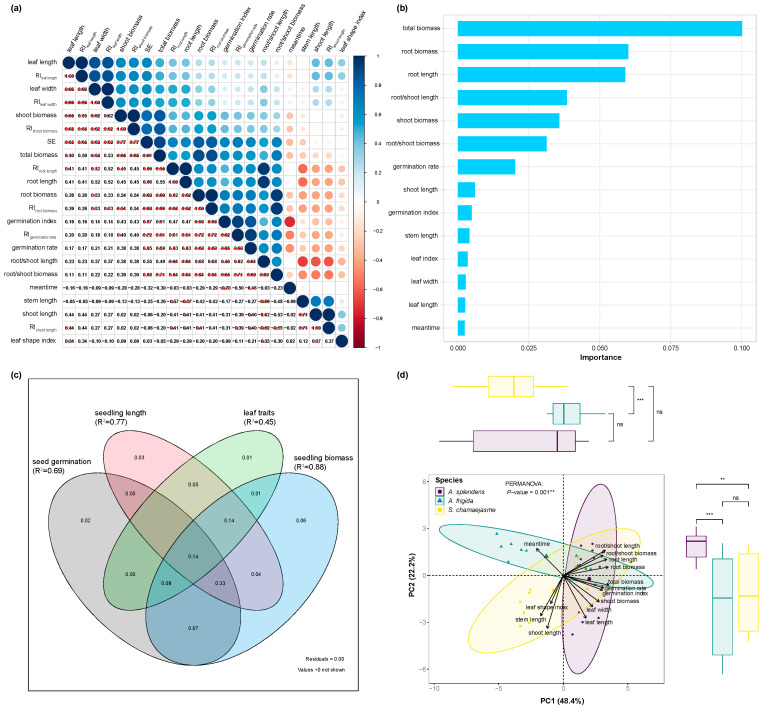
(**a**) Relationship between different parameters of germination and allelopathic index of lettuce. The red circle represents a negative relationship between parameters, and the blue circle represents a positive relationship between parameters. The larger the circle, the stronger is the correlation. The number represents the correlation coefficients calculated using the Spearman method. (**b**) The importance of phenotypic characteristics on SE by random forest analyses. (**c**) Variance partitioning analysis of four monitoring types (seed germination, seedling length, seedling biomass, and leaf traits). The number represents the explanatory power of monitoring types to SE. (**d**) Principal components analysis of seed germination and seedling growth of lettuce and the differences among three donor herbaceous species. ns: no significant difference, * *p* < 0.05, ** *p* < 0.01, *** *p* < 0.001.

A correlation analysis disclosed the relationships among lettuce seed germination, seedling growth, and the RI (Figure 6a). SE was strongly positively correlated with the total biomass, RI_root biomass_, root biomass, RI_shoot biomass_, shoot biomass, RI_germination rate_, root/shoot biomass ratio, germination index, RI_root length_, root length, germination rate, RI_leaf length_, leaf length, RI_leaf width_, and leaf width (Figure 6a, *p <* 0.05). SE was slight, albeit non significantly, negatively correlated with mean germination time, stem length, RI_shoot length_, and shoot length (Figure 6a, *p >* 0.05). Mean germination time, stem length, RI_shoot length_, shoot length, and leaf shape index were negatively correlated with other indices, while the other parameters were positively correlated with each other (Figure 6a). An RFA of the importance of the morphological characteristics on the SE demonstrated that total biomass was the most important characteristic followed by root biomass, root length, root/shoot length ratio, shoot biomass, root/shoot biomass ratio, and germination rate (Figure 6b). VPA showed that seedling biomass had the largest variance (R^2^ = 0.88) among all lettuce germination characteristics. The variances of seed germination, seedling length, and leaf traits were ~0.69, ~0.77, and ~0.45, respectively (Figure 6c). PCA revealed distinct differences between herbs in terms of their effects on lettuce germination performance (Figure 6d, *p <* 0.05). The first two principal components (PC1 and PC2) represented 48.4% and 22.2% of the variation in lettuce germination performance, respectively (Figure 6d). PC1 generally distributed the germination characteristics along with total biomass, root biomass, root length, root/shoot length ratio, germination rate, root/shoot biomass ratio, germination index, and shoot biomass (Figure 6d) and represented belowground plant performance. PC2 generally distributed the germination characteristics along with shoot length, leaf length, stem length, leaf width, leaf shape index, and mean germination time (Figure 6d) and represented aboveground plant performance. The differences between *A. frigida* and *S. chamaejasme* were significant in terms of the PC1 variables. However, the differences between *A. splendens* and the other two herbs were non-significant (Figure 6d). In contrast, the differences between *A. frigida* and *S. chamaejasme* were non-significant in terms of the PC2 variables. Nevertheless, the differences between *A. splendens* and the other two herbs were significant in terms of the PC2 variables (Figure 6d). After combining the results of the multivariate analyses, we concluded that seedling biomass, organ length, and seed germination rate are vital crucial germination indices in allelopathic effect assays. Furthermore, the SE calculated based on the five key indices was consistent with the original SE (Figure S2).

## 3. Discussion

### 3.1. Effects of Extract Concentration

Aqueous extracts simulate donor leachates and can be used to investigate allelopathic potential [31]. Seed germination rate determines individual survival and abundance. The germination index reflects seed germination in recipient plants [45]. Numerous studies reported that allelochemicals released by donor plants may significantly reduce seed germination and seedling growth in recipient plants in a concentration-dependent manner [23,34,35,36,37]. Here, we observed dose-dependent negative effects of *A. splendens*, *A. frigida,* and *S. chamaejasme* aqueous extracts on lettuce germination and root length. Therefore, lettuce growth fitness may be substantially attenuated in response to allelopathy mediated by the foregoing herbs. The allelochemicals may poison weak seeds, impede nutrient absorption, and hinder seedling growth and development [44]. On the contrary, the donor aqueous extracts had positive influences on lettuce shoot length and biomass as well as leaf dimensions. Our results contradict those of a previous report on the relatively weak negative effects of *A. frigida* aqueous extracts on shoot length [20]. The concentration range (0.025–0.15 g mL^−1^) used by Li et al. [20] may have been too high. Moreover, while Li et al. [20] recipients were monocotyledonous herbs, ours was a dicotyledonous crop. The results of our hormesis assay on lettuce seedling biomass reinforced those of previous findings, suggesting that low leachate concentrations of Canada goldenrod had positive effects in recipient germination and seedling growth at lower concentrations but negative effects at higher concentrations [34], especially at >0.055 g/mL [31]. At high concentrations, aqueous herbal extracts had allelopathic effects on lettuce seed germination capacity and uniformity, as well as the growth of belowground organs essential for competitive soil nutrient uptake and seedling growth. Moreover, aboveground organ growth is stimulated to increase photosynthesis and compensate for root growth inhibition.

### 3.2. Species-Specific Effects

It is well-known that plant species differ in allelopathic activity. [20,31,46]. Here, PCA revealed distinct differences among donor species in terms of lettuce germination (Figure 6d). Allelopathy is a potential succession mechanism to inhibit or promote component turnover [5,20,40,47]. *A.* *frigida* and *S. chamaejasme* are bioindicators of grassland degradation. Aqueous extracts of *A. frigida* and *S. chamaejasme* suppressed lettuce seed germination, inhibited root elongation, and had hormesis effects on seedling biomass and synthetical germination performance. Thus, allelopathic potential may partially account for the dominance of *A. frigida* and *S. chamaejasme* in degraded grasslands. *A.* *frigida* may allelopathically predominate in degraded grasslands by emitting volatile organic compounds (VOCs) [21,48] and excreting leaf leachates [20]. Umbelliferone is the main allelochemical of *S. chamaejasme,* and it enables the plant to invade natural terrestrial ecosystems [22,24]. Conversely, aqueous extracts of *A. splendens* significantly inhibited lettuce seed germination and root length by promoting lettuce seedling development. However, the synthetical germination performance was positive (Figure 5h). Thus, *A.* *splendens* may promote the growth of neighboring plants, and reseeding it may help conserve and restore degraded grassland.

Each receptor type may respond differently to donor extracts [49,50]. *L. sativa* is sensitive to allelopathic effects and is a model recipient plant in allelopathy bioassay studies [34,43,44]. However, *L. sativa* is not ecologically related to donor grassland plant species. To confirm the role of allelopathy in grassland plant community assembly, the allelopathic responses of ecologically related species must be evaluated [17]. Although some results have indicated a strong correlation between laboratory and field settings in terms of plant performance [51] and the effects of soil organisms on allelochemicals released by plants, such as transformation, degradation, and transport [1], there is still a need for a field-level study on the allelopathy. Therefore, we have arranged a pot experiment using a living species to study the allelopathy of degradation indicator plants on *L. chinensis* in 2021.

### 3.3. Vital Germination Characteristics

Here, aqueous extracts of donor plants significantly suppressed lettuce seed germination, whereas the responses of seedling development to aqueous extracts of donor plants varied among plant organs. Our morphological results confirmed that aqueous extracts of donor plants affected root growth more strongly than shoot growth, thus reinforcing previous conclusions [52,53]. Generally, lettuce shoot growth parameters increased, while those of the roots decreased in response to aqueous plant extracts. Root and shoot length and biomass ratio decreased in response to aqueous donor plant extracts. Petri dish assays revealed that lettuce seedling roots turned dark and failed to grow gravitropically in response to high aqueous donor plant extract concentrations (Figure S1). RFA of the relative importance of phenotypic traits on the SE indicated that root performance ranked ahead of shoot performance (Figure 6b). Hence, PC1 represents belowground performance, whereas PC2 represents aboveground performance (Figure 6d). Therefore, the root was more sensitive to allelochemicals than the shoot. The root is the main organ regulating plant water and nutrient absorption and can resist stress and adverse habitats. Nevertheless, the sensitivity of roots to allelopathy underscores the utility of seedling roots in allelopathy detection. Root diameter (RD) and specific root length (SRL) convey a trade-off between root lifespan and resource foraging efficiency [54]. Plants may inhibit root elongation and promote root thickening under low-level allelopathic stress. In the present study, root biomass increased under this condition and helped to extend root longevity. In contrast, gains in root biomass are inhibited under severe allelopathic stress. In this state, both resource foraging and root lifespan are reduced. Allelopathy is a defensive mechanism in response to strong selection pressure associated with competition. Furthermore, allelopathy helps avoid competitive interactions and is an aggressive strategy that weakens the performance of proximate competitors [21]. During plant degradation, *A. frigida* and *S. chamaejasme* have a competitive advantage by releasing allelochemicals into the soil in response to rain and fog, thereby inducing stress in neighboring plants. Recipient plant roots avoid toxicity by undergoing growth inhibition.

Allelopathy had a stronger negative effect on seed germination than on seedling growth. However, previous studies reported that leachate allelopathy had exactly the opposite effects [31]. There may have been species-specific (donor and recipient) and concentration-specific differences among previous studies. Our results showed that the herbs predominating in degraded grassland curtail the population sizes of competing plant species, by inhibiting seed germination in the latter. Certain competing plants may nonetheless survive, as they are strongly competitive during seed germination.

Correlation analyses, RFA, VPA, and PCA confirmed that seedling biomass is a critical parameter in allelopathy detection (Figure 6). However, other studies used germination rate [38,39,42] or root length [34,40,55] to describe recipient plant performance. Few studies have also monitored seedling biomass [34,56] to detect allelopathy. Biomass is the accumulation of dry matter, which is an intuitive evaluation of plant growth. By combining the results of our multivariate analyses, we propose that seedling biomass, organ length, and seed germination rate may be the most important allelopathy indices. The SE calculated by these five key indicators was consistent with the original SE (Figure S2). Therefore, these indices may effectively represent germination performance while assessing allelopathic potential.

## 4. Materials and Methods

### 4.1. Plant Materials

Lettuce (*L. sativa*) was selected as the recipient plant, while three grassland herbs (*Artemisia* *frigida*, *Stellera* *chamaejasme*, and *Achnatherum splendens*) were chosen as donor plants. Lettuce seeds were purchased from an online agricultural supplier (Seed industry of Hezhiyuan, Shandong Province, China, https://shop58479028.taobao.com/?spm=a230r.1.0.0.43744decvZGoxZ, accessed on 20 November 2021). In August 2020, fully expanded, mature, undamaged shoot (aboveground) samples of the donor plants were randomly collected from the National Grassland Ecosystem Field Station, Guyuan County, Hebei Province, China (41°46′ N, 116°14′ E, 1460 m a.s.l.), in the southeastern corner of the Inner Mongolia steppe. Chinese rye grass (*Leymus chinensis*) is the dominant plant species of the steppe. However, with long-term overgrazing by sheep and cattle, *Artemisia frigida* and *S. chamaejasme* replaced *L. chinensis* as the dominant species of degraded grassland in the region. Meanwhile, *Achnatherum splendens* has not changed significantly as a subdominant species.

### 4.2. Allelopathy Bioassays

Aqueous extracts of air-dried donor plant shoots were used to simulate plant leachates that form under rain and fog [34,38]. The extracts were prepared in duplicate from 40 g air-dried ground shoots tissue in 400 mL distilled water at a constant 20 °C in a dark incubator for 12 h and 24 h. The material was filtered optimally as follows: two layers of cheesecloth were used to remove particulate matter and the filtrate was vacuum-filtered through 10 μm and 0.45 μm membranes to remove most microorganisms. The filtered liquids served as the initial aqueous extracts and were stored at −20 °C to minimize contamination and deterioration. Each initial extract was diluted to 0.05 g mL^−1^ (high concentration, HC) and 0.005 g mL^−1^ (low concentration, LC) with distilled water and stored at 4 °C until and during use [38,43].

To assess the allelopathic potential of each donor plant species, three replicates were prepared with two different soaking times and extract concentrations, with distilled water used as the control (CK). All lettuce seeds were surface-sterilized with 1% (*v/v*) sodium hypochlorite (NaClO) for 15 min and rinsed with sterile distilled water (*d*H_2_O) [34]. For each treatment, 30 healthy sterile seeds were placed in 9 cm Petri dishes containing two layers of filter papers soaked with either 5 mL aqueous extract or *d*H_2_O. The dishes were then set in an artificial light incubator (GXZ-430B model, Ningbo Jiangnan Instrument Factory, Ningbo, China; 20 °C; 8 h/16 h light/dark cycle; 12,000 lx). Two milliliters of aqueous extract or *d*H_2_O were added to each dish daily.

### 4.3. Allelopathic Potential Determination

Germinated seeds were enumerated daily. Lettuce seeds were scored as germinated if their radicle length was 1–2 mm. The germination rate was calculated as the ratio of the final number of germinated seeds to the total number of seeds used. The germination index was calculated as ΣGi/Ti [57,58]. The mean germination time was calculated as ΣGiTi/ΣGi, where Gi is the number of radicles emerging at time I, and Ti is the number of days after planting [20].

After 7 d, 10 robust seedlings per Petri dish were randomly selected to estimate seedling growth. The seedling length was the sum of the shoot and root lengths. The root/shoot length ratios were calculated. The stem length was the shoot length minus the leaf length. The seedling biomass was the sum of the shoot and root biomass and was determined after the tissues were dried at 65 °C for 72 h. The root/shoot biomass ratio was calculated, and the total biomass was the shoot biomass plus the root biomass. The leaf phenotype comprised the leaf length and width. The leaf shape index was the ratio of the leaf length to the leaf width [59,60].

The allelopathy response index (RI) was evaluated to determine the allelopathic effects of the aqueous grassland plant extracts on lettuce seed germination and seedling growth. The RI was defined as 1-C/T if T ≥ C or as T/C-1 if T < C, where C and T are the control and treatment values, respectively [61]. The synthetical allelopathic effect index (SE) described the synthesized impact of each aqueous herb extract on *L. sativa* germination and was calculated as the arithmetic average of the RI of the test items under the same treatment [41,62]. Here, SE = (RI_GR_ + RI_SL_ + RI_RL_ + RI_LL_ + RI_LW_ + RI_SB_ + RI_RB_)/7, where GR is the germination rate, SL and RL are the shoot and root lengths, respectively, LL and LW are the leaf length and width, respectively, and SB and RB are the shoot and root biomass, respectively.

### 4.4. Statistical Analyses

All variables were pre-tested for normality and homogeneity of variance. Non-parametric tests were used when the assumptions of normality and homogeneity of variance were not met. A three-way ANOVA permutation test was used to assess seed germination and seedling growth among species, extract concentrations, soaking times, and their interactions. Wilcoxon’s rank sum test was used to determine the significance of soaking time. Differences among extract concentrations, in terms of their effects on lettuce seed germination and seedling growth, were assessed by Tukey’s multiple-range permutation test. Statistically significant differences were set at *p* < 0.05. Data were scaled prior to multivariate analysis. Spearman’s correlation analysis was used to clarify the relationships among indices. A random forest analysis (RFA) was conducted to establish the importance of seed germination and seedling growth on SE. A variance partitioning analysis (VPA) was performed to compare variance among seed germination, seedling length, seedling biomass, and leaf traits. A principal component analysis (PCA) was conducted to determine the axes of variation among the germination characteristics. PERMANOVA was applied to assess the differences among the donor plant species. All statistical analyses and graph plotting were performed in R v. 4.0.4 (R Core Team, 2021).

## 5. Conclusions

Aqueous extracts of three donor plants (*A. splendens*, *A. frigida*, and *S. chamaejasme*) inhibited *L. sativa* seed germination and seedling root length but promoted seedling shoot length and biomass as well as leaf size. Aqueous extracts of *A. splendens* promoted seedling root biomass and synthetical allelopathic effect (SE), whereas *A.* *frigida* and *S. chamaejasme* had hormesis effects. The negative allelopathic results of *A. frigida* and *S. chamaejasme* may contributed to grassland degradation. Reseeding allelopathy-promoting species such as *A. splendens* may be beneficial to grassland restoration. After combining the foregoing results with those of the multivariate analyses, we concluded that seedling biomass, root and shoot length, and seed germination rate are best suited as representative bioindicators of allelopathy in lettuce. Finally, extract soaking times (12 h or 24 h) did not exhibit significantly different effects on lettuce seed germination or seedling development. However, there is still a need for a field-level study on the allelopathy of living degradation indicator plants such as *Leymus chinensis*.

## Data Availability

Not applicable.

## Supplementary Materials

The following supporting information can be downloaded at: https://www.mdpi.com/article/10.3390/plants11040486/s1, Figure S1: Photos of 4 donor plants aqueous extracts with different concentrations and soaking time on lettuce seed germination on the last day of the count; Figure S2: Effects of extract concentration and soaking time of three donor species-*A. splendens*, *A. frigida*, and *S. chamaejasme* on lettuce SE. SE = (RI_GR_ + RI_SL_ + RI_RL_ + RI_SB_ + RI_RB_)/5.
