# Supplementary material for: Aqueous Extracts of Three Herbs Allelopathically Inhibit Lettuce Germination but Promote Seedling Growth at Low Concentrations
Source: Plants (Basel). 2022 Feb 11;11(4):486. doi: 10.3390/plants11040486 (PMC8877897; doi:10.3390/plants11040486)
Supplement: Supplementary file 1 [file plants-11-00486-s001.zip › plants-1589410-supplementary.pdf]

Supplementary Material

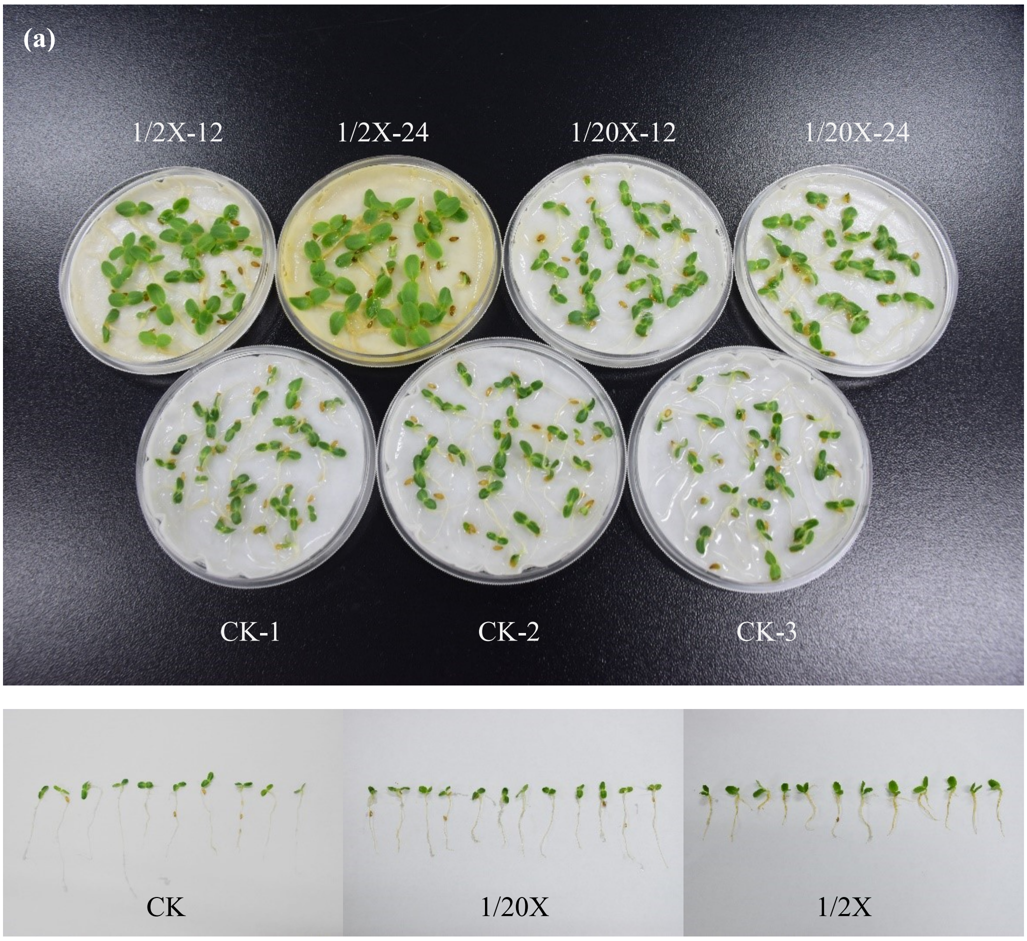

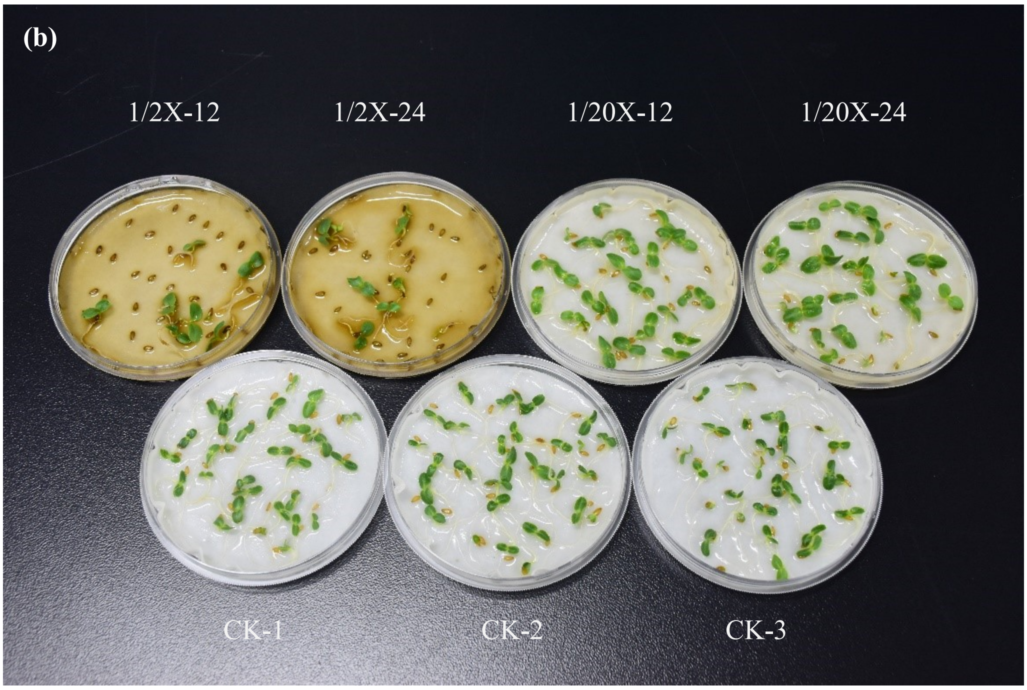

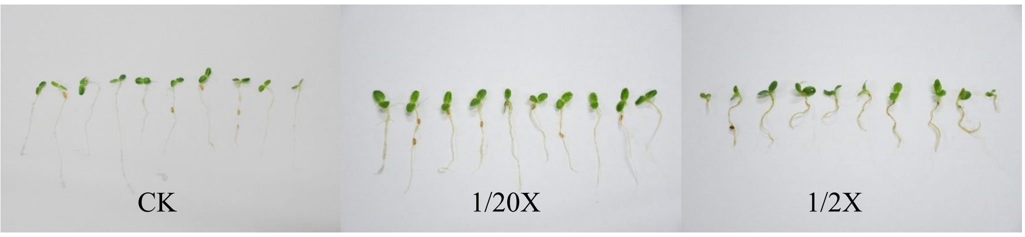

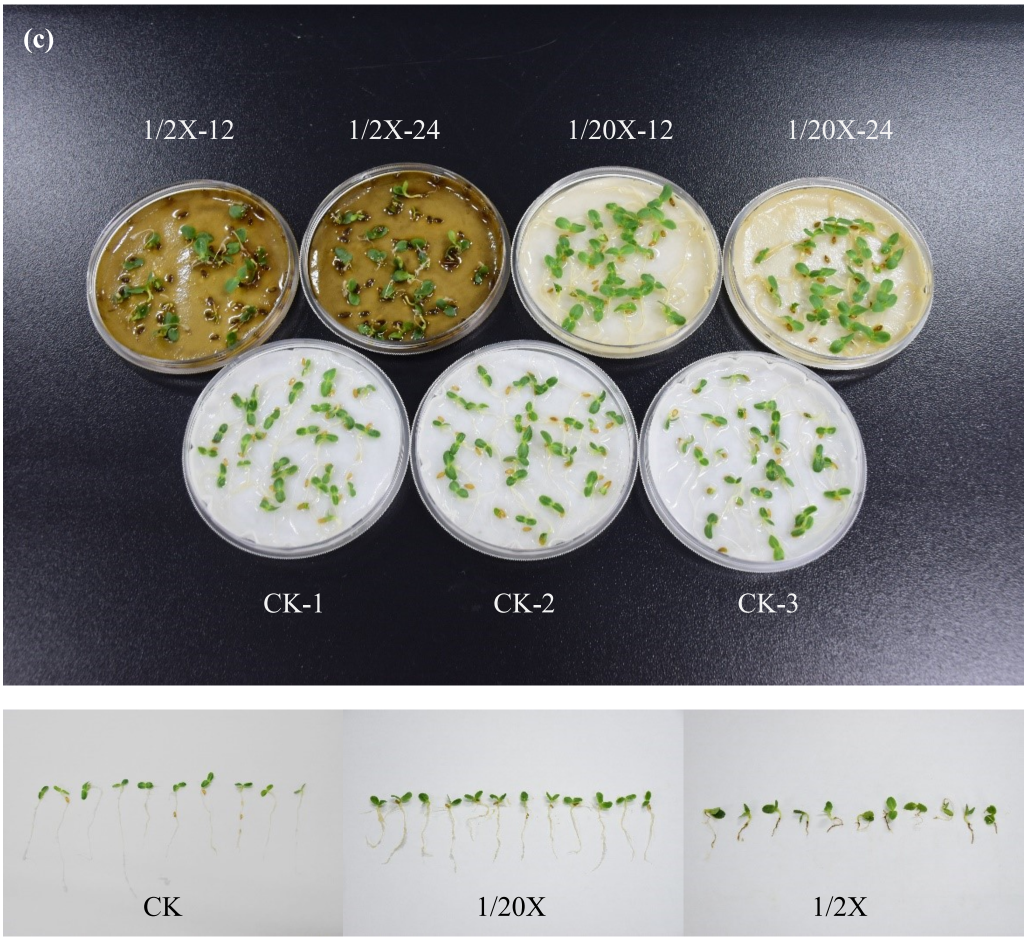

Figure S1. Photos of 4 donor plants aqueous extracts with different concentrations and soaking time on lettuce seed germination on the last day of the count. (a) *Achnatherum splendens*. (b) *Artemisia frigida*. (c) *Stellera chamaejasme*. 1/2X-12 represents extract concentration at 0.05 g mL^{-1} and soaking time about 12 h; 1/20X-24 represents extract concentration at 0.005 g mL^{-1} and soaking time about 24 h. The arranged seedlings were those under aqueous extract about 12 h soaking time.

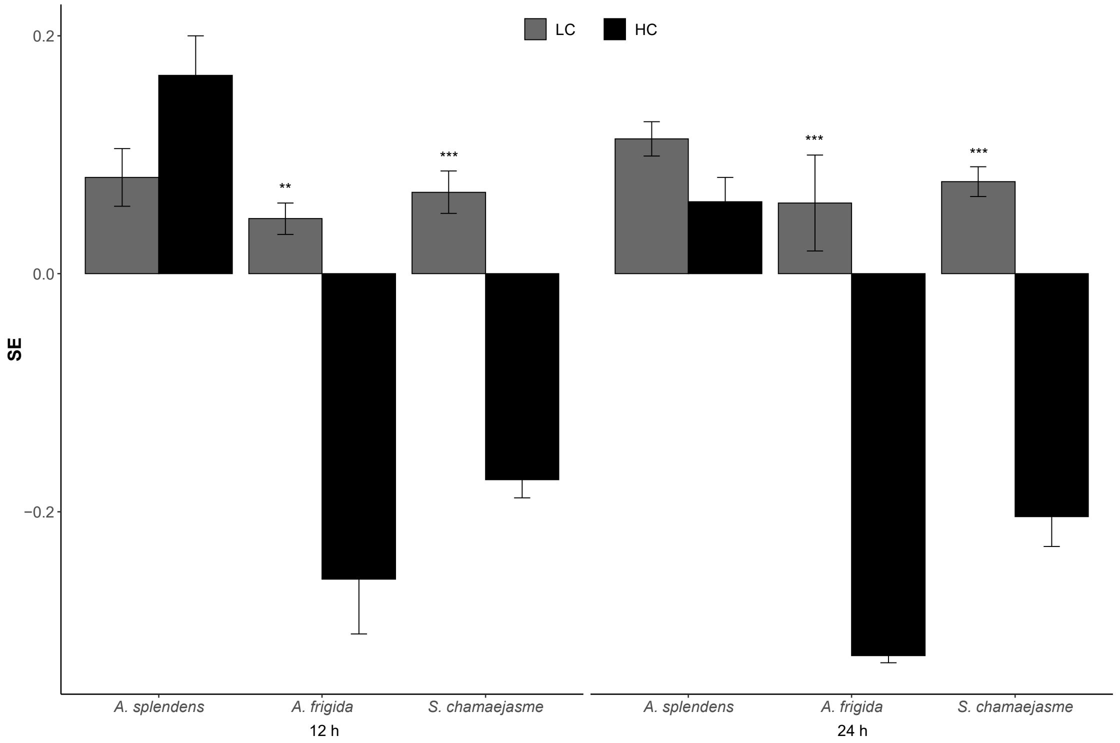

Figure S2. Effects of extract concentration and soaking time of three donor species-*A. splendens*, *A. frigida*, and *S. chamaejasme* on lettuce SE. SE = $(RI_{GR}+RI_{SL}+RI_{RL}+RI_{SB}+RI_{RB})/5$. Data are presented as mean ± SE (*n*=36). LC, low concentration (0.005 g mL-1). HC, high concentration (0.05 g mL-1). S, donor species. C, extract concentration. T, soaking time. *P<0.05, **P<0.01, ***P<0.001. There were no significant differences between the two soaking times with the Wilcoxon rank sum test (P>0.05).
